# Mechanistic and Pharmacological Issues of Aspirin as an Anticancer Agent

**DOI:** 10.3390/ph5121346

**Published:** 2012-12-05

**Authors:** Melania Dovizio, Stefania Tacconelli, Carlos Sostres, Emanuela Ricciotti, Paola Patrignani

**Affiliations:** 1 Department of Neuroscience and Imaging, Center of Excellence on Aging (CeSI), “G. d’Annunzio” University, Via dei Vestini 31, 66100 Chieti, Italy; E-Mail: m.dovizio@unich.it (M.D.); s.tacconelli@unich.it (S.T.);ppatrignani@unich.it (P.P.); 2 University of Zaragoza School of Medicine, University Hospital Lozano Blesa, IIS Aragón. CIBERehd, 50009 Zaragoza, Spain; E-Mail: carlossostres@gmail.com(C.S.); 3 Institute for Translational Medicine and Therapeutics, University of Pennsylvania, Philadelphia, PA 19104, USA; E-Mail: emanuela@mail.med.upenn.edu (E.R)

**Keywords:** aspirin, colorectal cancer, platelet, cycloooxygenase-1

## Abstract

Recent findings have shown that aspirin, taken for several years, reduces the long-term risk of some cancers, particularly colorectal cancer. The result that aspirin benefit is detectable at daily low-doses (at least 75mg), the same used for the prevention of cardiovascular disease, positions the antiplatelet action of aspirin at the center of its antitumor efficacy. At low-doses given every 24 h, aspirin is acting by a complete and persistent inhibition of cyclooxygenase (COX)-1 in platelets (in the pre-systemic circulation) while causing a limited and rapidly reversible inhibitory effect on COX-2 and/or COX-1 expressed in nucleated cells. Aspirin has a short half-life in human circulation (approximately 20 min); nucleated cells have the ability to resynthesize the acetylated COX-isozymes within a few hours, while platelets do not. COX-independent mechanisms of aspirin, such as the inhibition of Wnt/ β-catenin and NF-kB signaling and the acetylation of extra-COX proteins, have been suggested to play a role in its chemo-preventive effects, but their relevance remains to be demonstrated *in vivo* at clinical doses. In conclusion, the results of clinical pharmacology and the analysis of randomized and epidemiological studies suggest that colorectal cancer and atherothrombosis share a common mechanism of disease, *i.e.* enhanced platelet activation in response to injury at distinct sites.

## 1. Introduction

A large body of clinical evidence indicates that aspirin can protect from different types of cancer, in particular colorectal cancer [1]. A role for the antiplatelet effect of aspirin in its anti-cancer effects is supported by several lines of evidence. A convincing indirect proof is one of the cardiovascular randomized clinical trials (RCT)s, the Thrombosis Prevention Trial [2], in which the chemopreventive effect of aspirin was detected on long-term follow-up [3,4], that involved the administration of a controlled-release formulation of aspirin (75 mg) with negligible systemic bioavailability [5], thus acting almost exclusively by affecting cyclooxygenase (COX)-1-dependent platelet function [6]. Another piece of evidence to sustain the notion that aspirin acts as an antitumor agent through the inhibition of platelet activation derives from the finding that a maximal chemopreventive effect is detectable at low-doses given once daily, in long-term analyses of cardiovascular RCTs and RCTs of adenoma recurrence [7,8,9,10], as well as in the vast majority of observational studies performed in different settings and with different methodology [11]. A similar dose-response has been shown for the antithrombotic effects of aspirin in clinical studies [12]. At the same doses (75-100 mg once daily), aspirin causes an almost complete inhibition of platelet COX-1 activity [6]. Altogether these findings show that the maximal inhibitory effects of low-dose aspirin in inhibiting COX-1-dependent platelet function roughly coincides with its clinical benefit in reducing the risk of cardiovascular and cancer clinical outcomes. This finding is convincing evidence, even if indirect, to support the hypothesis that the antiplatelet effect of aspirin is a central mechanism for the prevention of vascular events and tumorigenesis.

In this review we will discuss the clinical results related to the impact of aspirin on the risk of cancer. Then, we will explain in detail the pharmacology of aspirin at low and high doses in order to give a mechanistic interpretation of aspirin action as a chemopreventive agent for cancer. In particular this review will enlighten the possible role of enhanced platelet activation in response to endothelial and epithelial injury in cellular transformation. We hypothesize that low-dose aspirin may affect relatively early events in the progression from normal mucosa to adenoma by selectively blocking platelet COX-1 activity and in turn platelet-dependent induction of cellular transformation (possibly mediated by upregulation of COX-2 in stromal cells). Higher doses of aspirin might also directly affect COX-2 activity once it is expressed in intestinal adenomas and this may translate into reduction of advanced lesion progression and metastasis. The proposal that platelet activation is a causal event of both cancer and cardiovascular disease is further supported by our recent results of enhanced systemic biosynthesis of TXA_2_, mainly from platelet COX-1, in colorectal cancer and in familial adenomatous polyposis (FAP), a colon cancer syndrome caused by mutations in one of two genes: APC and MUTYH [13,14].

## 2. Aspirin and Colorectal Cancer Risk: the Results of Clinical Studies

Colorectal cancer [CRC) is one of the most common malignancies in the World. According to a recent report, it is the third most common cancer in men (663,000 new cases per year, 10.0% of the total) and the second in women (570,000 new cases per year, 9.4% of the total). About 608,000 deaths from CRC were estimated worldwide in 2008, accounting for 8% of all cancer deaths, making it the fourth most common cause of death from cancer [15]. Screening reduces CRC mortality and is recommended beginning at age 50 for average risk individuals, although compliance is far from adequate and not widely available in resource-poor settings [16,17]. Therefore, understanding the action of new chemoprotective agents, such as aspirin, remains highly necessary.

There is large body of evidence from epidemiologic observations and population-based studies that aspirin and other nonsteroidal antiinflammatory drugs (NSAIDs) have a chemopreventive effect on several cancer types, CRC being among them. The protective effect of NSAIDs has been underscored in most epidemiologic studies, showing prevention of adenoma recurrence and reduction of CRC incidence and mortality. However, the protective effect appears to depend on the type of drug, dose and mainly the duration of exposure [18,19,20,21,22].

Aspirin has become the most likely NSAID for chemopreventative use because of its known cardiovascular (CV) benefits and available efficacy and safety data. Even so, the US Preventive Services Task Force (USPTF) does not recommend aspirin for primary chemoprevention, although it has highlighted the need for establishing the age at which treatment should be started, the most effective duration of intake and the optimum dosage [23].

First, epidemiological studies have provided information on the chemopreventive effect of aspirin against CRC. In fact, both case-control and cohort studies have found that aspirin use (regular and daily) was associated with approximately 50% reduction in the incidence and mortality of CRC, both in men and women [1]. However, this benefit was observed after at least ten years of regular aspirin use [4].

Recently, Rothwell *et al.* linked data on cancer outcomes to RCTs originally designed to examine the effect of aspirin on CV disease prevention [3]. Included trials examined both patient populations at low CV risk (n = 10,224) [2,24], as well as individuals with higher risk (history of transient ischaemic attack, minor stroke, or retinal artery occlusion, n = 3809) [2,24]. They found that treatment with aspirin doses between 75 and 500 mg/day reduced the 20–year risk of CRC by 24% and associated mortality by 35%, with increasing benefit observed with longer durations of treatment. There was a suggestion that the reduction in CRC incidence may be largely confined to the proximal colon compared with the distal colon (p for difference = 0.04). The main limitation of this analysis is that the included studies were CV prevention trials not originally designed to examine cancer incidence or mortality.

Another study by Rothwell *et al.* examined the effects of aspirin treatment on mortality due to all cancers [4], including data from eight CV–prevention RCTs of daily aspirin. Among the eight trials with a total of 25,570 patients and 674 cancer–related deaths during the trial periods, aspirin treatment at a dose ranging from 75 to 1200 mg/day was associated with a reduction of 21% risk of death from any cancer. Benefit was observed only after five years of follow–up. Among the six trials with data on the specific site of cancer occurrence, patients randomized to aspirin had a reduced risk of death due to CRC (HR, 0.41; 95% CI, 0.17–1.00, p=0.05), beginning five years after the initiation of aspirin treatment. Finally, Last, in the two largest RCTs performed on healthy individuals [The Physicians’s Health (PHS) and Women’s Health (WHS) Studies], aspirin (325 and 100 mg, respectively) did not significantly reduce the risk of CRC [25,26]. The absence of aspirin effect found in these RCTs might be explained by some limitations in the design of the trials, *i.e.* inadequate treatment duration or follow-up (in PHS), equivalent dose of aspirin 50 mg/day (in the WHS) lower than the 75 mg/day shown to be effective in both meta-analyses and/or administration schedule (in WHS the drug was given every other day).

The benefit of aspirin use in CRC prevention has also been extrapolated from RCTs of aspirin in the prevention of adenomas (Table 1), the precursor lesions of the majority of CRC. Adenomas can be a useful surrogate end–point for CRC prevention since their development is considerably shorter than the evolution of CRC, which is believed to require between 5–10 years. Until now, four different multicenter, randomized controlled trials assessing the efficacy of aspirin in the moderate-risk population have been identified [7,8,9,10]. A meta-analysis of data from these four studies [27] showed a significant 21% reduction in the risk for recurrence of any type of adenoma (RR, 0.79; 95% CI, 0.68 to 0.92; p=0.002), with a moderate level of heterogeneity among the studies (34%). In addition, there was a 7% reduction in the absolute risk for recurrence (risk difference [RD], 0.07; 95% CI, 0.11 to 0.04; p<0.0001), with no statistical heterogeneity. When the analysis was limited to the comparison of aspirin with placebo alone (without folic acid), the risk for adenoma recurrence remained similar (RR, 0.80, 95% CI, 0.65 to 0.98; p = 0.03), with a moderate level of heterogeneity (47%). Logan *et al.* [10] and Cole *et al.* [28] compared aspirin plus folic acid with placebo and they did not find significant differences in the relative and absolute risk for recurrence of any type of adenoma, with no statistical heterogeneity between these studies. A possible interference of folic acid on the chemopreventive effect of aspirin might explain these results and this hypothesis should be investigated in further studies. The meta-analysis by Cole *et al.* [27] included also the results after one year of follow–up in one of the RCTs, the Association pour la Prevention par l’Aspirine du Cancer Colorectal Study Group [APACC) Trial, which randomized 272 patients with colorectal adenomas to receive soluble lysine acetylsalicylate 160 mg/day, 300 mg/day or placebo. Treatment with either dose of aspirin was associated with a significant reduction in risk of recurrent adenoma at one year [9]. However, no statistically significant reduction in adenoma recurrence was demonstrated after four years follow-up [29]. Additional studies are ongoing, including the Japan Colorectal Aspirin Polyps Prevention (JCAPP)study, a RCT examining the effect of aspirin 100 mg/day on occurrence of recurrent tumors two years after endoscopic removal of colorectal adenomas and cancers in a Japanese population [30].

Chemopreventive effect of aspirin in FAP patients have been also assessed, but limited information is now available. In one study performed by Burn *et al.* [31], a randomized, placebo–controlled trial of aspirin (600 mg/day) and/or resistant starch (RS, 30 g/day) (CAPP1) performed in FAP patients from 10 to 21 years old, the daily administration of aspirin 600 mg from one to 12 years did not realize the primary end–point consisting in the reduction of polyp number in the rectum and sigmoid colon. In contrast, the drug treatment significantly reduced the size of polyps. These data are promising but we have to take care about several limitations, such as a lack of standardization of the extent of endoscopic examination and surveillance done by multiple endoscopists at 12 different treatment centres [31,32]. 

Table 1Randomized clinical trials of aspirin in the chemoprevention of colorectal neoplasia.

**(a):** Clinical Trials of Sporadic Colorectal Adenoma.

**Study** (reference) Patients	Treatment	Primary end-point	Relative risk (RR) (95% Confidence Interval, CI)
**AFPPS trial **[7,28]: Patients with a recent history of histologically documented (removed) adenomas	Aspirin (81 mg or 325 mg daily) or folic acid (1 mg daily) or placebo for 2.7 years	Proportion of patients in whom one or more colorectal adenomas were detected by colonoscopy	Any adenoma: RR: 0.81 (0.69–0.96), aspirin 81mg * versus* non aspirin RR: 0.96 (0.81–1.13), aspirin 325 mg* versus* non aspirin RR: 1.04(0.90–1.20), folic acid* versus* non folic acid Advanced lesion: RR: 0.59 (0.38–0.92), aspirin 81mg* versus* non aspirin RR: 0.83 (0.55–1.23), aspirin 325 mg* versus* non aspirin RR: 1.32 (0.90–1.92), folic acid* versus* non folic acid
**CAPS trial** [8]: Patients with a histologically documented colon or rectal cancer with a low risk of recurrent disease	Placebo or enteric coated aspirin 325 mg daily for 2.6 years	Detection of adenomas in the large bowel by either colonoscopy or sigmoidoscopy	RR: 0.65(0.46–0.91)
**APACC trial** [9,29]: Patients with a history of colorectal adenomas	Placebo or lysine acetylsalicylate (160 or 300 mg daily) for 1 and 4 years	Proportions of recurrent adenomas and adenomatous polyp burden by colonoscopy	RR: 0.73 (0.52–1.04) for both doses, after 1 year RR: 0.96(0.75–1.22), for both doses, after 4 years
**ukCAP trial** [10]: Patients with an adenoma removed in the 6 months before recruitment	Enteric coated aspirin (300 mg daily) plus placebo or aspirin plus folic acid (0.5 mg/daily) or folic acid plus placebo or double placebo for about 2.6 years	Percentage of patients who developed one or more recurrent colorectal adenomas or cancers by colonoscopy	Any adenoma: RR: 0.79(0.63–0.99), aspirin* versus* non aspirin RR: 1.07 (0.85–1.34), folic acid* versus* non folic acid Advanced adenoma: RR: 0.63(0.43–0.91), aspirin* versus* non aspirin RR: 0.98(0.68–1.40), folic acid* versus* non folic acid
**J-CAPP trial** [30]: Patients with previous sporadic colorectal tumors	Enteric coated aspirin (100 mg daily) or placebo for 2 years	Presence or absence of new colorectal tumors by colonoscopy	Ongoing

AFPPS: Aspirin/Folate Polyp Prevention Study; CAPS: Colorectal Adenoma Prevention Study; APACC: Prévention par l'Aspirine du Cancer Colorectal; ukCAP: United Kingdom Colorectal Adenoma Prevention; J-CAPP: Japan Colorectal Aspirin Polyps Prevention.

**(b):** Clinical Trials of Hereditary Colorectal Neoplasia

**Study** (reference) Patients	Treatment	Primary end-point	RR or Hazard ratio (HR) (95% CI)
**CAPP 2 trial** [35]: Lynch syndrome (hereditary non-polyposis colon cancer or HNPCC)	Aspirin (600 mg daily) or aspirin placebo or resistant starch (30g daily) or starch placebo, for up to 4 years	Detection of at least one adenoma or colorectal carcinoma by colonoscopy	HR: 0.63 (0.35–1.13), for the entire post-randomization period (aspirin* versus* placebo) HR: 0.41(0.19–0.86), for ≥2years of treatment (aspirin* versus* placebo)
**CAPP1 trial** [31]: FAP young patients (10 to 21 years of age)	Aspirin (600 mg daily) plus placebo or resistant starch (30 g daily) plus placebo or double placebo for 17 years	Polyp number in the rectum and sigmoid colon by colonoscopy	RR: 0.77 (0.54–1.10), aspirin* versus* non aspirin RR : 1.05(0.73–1.49), resistant starch* versus* non resistant starch
**J-FAPP II trial** [30]: FAP patients (≥16 years of age)	Placebo* versus* enteric coated aspirin (100 mg daily for 6-10 months)	Reduction in the number of rectal tumors	Ongoing

CAPP: Colorectal Adenoma/Carcinoma Prevention Programme; J-FAPP: Japan Familial Adenomatous Polyposis Prevention

The efficacy of lower doses of aspirin (100 mg/day) in FAP patients is currently being explored in the Japan Familial Adenomatous Polyposis Prevention II (J-FAPP II) trial [30]. With respect to the incidence of CRC, whereas the Colorectal Adenoma/Carcinoma Prevention Programme (CAPP)1 study did not report data on this outcome, the CAPP 2 study (the first aspirin study to have CRC prevention as a primary end-point) showed that aspirin use was not associated with a statistically significant reduction in the risk of developing CRC after 2.5 years of follow-up (RR, 0.87; 95% CI, 0.39 to 1.96; p=0.74) [33,34]. However, aspirin seemed to delay the appearance of new cancers in patients with longer treatment durations. In this issue, the long-term follow-up of participants randomly assigned to aspirin or placebo in the CAPP 2 trial has been recently reported [35]. Whereas in the intention-to-treat analysis of time to first CRC there was no statistically significant difference (HR, 0.63; 95%CI, 0.35 to1.13; p=0.12), the *per*–protocol analysis in participants completing two years of intervention resulted in an HR of 0.41 (95%CI, 0.19 to 0.86; p=0.02) (Table 1). Importantly, adverse events did not differ between aspirin and placebo groups, although it should be taken into account that no data for adverse events were available for the post-intervention period (*e.g*. anaemia associated with aspirin use) [35].

### 2.1. Chemopreventive Effects of Aspirin in All Cancers

As reported above, one recent study by Rothwell *et al.* [4] showed that aspirin reduced risk of death due to cancer by about 20% in the CV trials, due mainly to a 34% reduction in cancer deaths after five years (even at doses of 75 mg/day). By long-term post-trial follow-up of patients in three of these trials, they showed that the 20-year risk of cancer death remained about 20% lower in the aspirin groups, and that benefit increased with scheduled duration of treatment in the original trial. The latent period before an effect on deaths was about five years for oesophageal, pancreatic, brain and lung cancer, but was more delayed for stomach, colorectal, and prostate cancer. For lung and oesophageal cancer, benefit was confined to adenocarcinomas. These results therefore provide the first reliable evidence that aspirin prevents non-colorectal cancer in humans, which is consistent with previous predictions of effects on cancers of the oesophagus, stomach, pancreas, lung, prostate and possibly brain [36,37]. The estimate of the effect of aspirin on death due to cancer in the first five years of the trials does not exclude a clinically important short-term benefit for cancers. Rothwell *et al.* [38] studied cancer deaths in all trials of daily aspirin *versus* control and the time-course of the effects of low-dose aspirin on cancer incidence and other outcomes in trials in primary prevention. Using individual patient data from all randomised trials of daily low-dose aspirin in primary prevention of vascular events, they showed that aspirin also reduces cancer incidence, both in men and women and in smokers and non-smokers. The effects of aspirin on other major outcomes (i.e. risk of major vascular events and the increase in risk of major extracranial bleeding) evolve with duration of treatment. Interestingly, the reduced risk of cancer is the only which is statistically significant from three years onwards. In addition, recently Rothwell *et al.* [39] found that the aspirin prevention of distant metastasis could account for the early reduction in cancer deaths in trials of daily aspirin *versus* control. This finding suggests that aspirin might help in treatment of some cancers and provides proof of principle for pharmacological intervention specifically to prevent distant metastasis.

Altogether the results of the analyses of Rothwell *et al.* [3,4,38,39] suggest that in terms of preventing spread of cancer, the chemopreventive effect of aspirin is largest in adenocarcinomas. These include cancers of the gut, particularly colorectal cancer, some cancers of the lung and most cancers of the breast and prostate. In terms of preventing the longer-term development of new cancers, the largest reductions are seen in risk of colorectal cancer and oesophageal cancer, with smaller effects on several other common cancers.

### 2.2. Balancing Risk and Benefits

**Figure 1:**
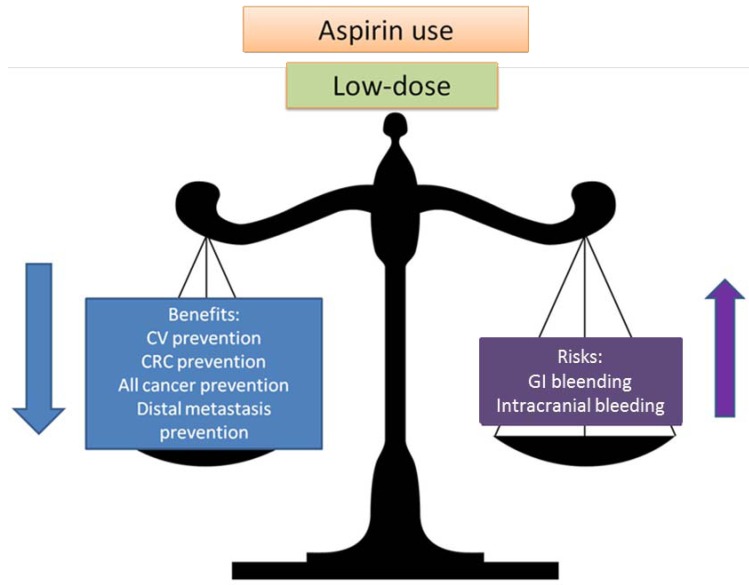
Balancing risks and benefits of aspirin use.

On the basis of current evidence, it can be assumed that low-dose aspirin taken over a 5-year period in patients at risk of CV events will probably prevent between 12 and 40 myocardial infarctions *per* 1,000 patients treated, assuming an overall 10% risk of CV events in this population of patients [40]. Conversely, the treatment is probably associated with a risk of 2–4 upper gastrointestinal bleeds per 1,000 patients treated [40]. However, it should be considered that the risk of upper gastrointestinal bleeding (and also CV events) changes in different individuals based on the presence of risk factors, such as the patient’s age, gender and pre-existing gastrointestinal ulcer history. Therefore, in specific patients, the benefits of aspirin will outweigh the risk of cardiovascular events if we reduce the gastrointestinal risk. Although the relative (RR, 1.43; 95% CI, 0.85–2.42) and absolute (1–2 intracranial bleeds per 10,000 patient-years) risk of intracranial bleeding with low-dose aspirin use is lower than the corresponding risk of GI bleeding [41,42], the generally more severe consequences of intracranial bleeding do weight heavily in overall considerations of risk and benefit. Given the risk of bleeding, clinical guidelines as the United States Preventative Services Task Force (USPSTF) recommended against the routine use of aspirin for CRC prevention in average-risk individuals in 2007 [23]. However, such risk–benefit calculations might require reconsideration based on the recent evidence supporting a benefit of daily aspirin use in the prevention of death from several cancers, including those from the GI tract [4], prevention of distal metastasis [39] and no increase of gastrointestinal toxicity risk of aspirin with prolonged use [43]. Therefore, the accumulating data from randomized clinical trials provide an exciting opportunity to reconsider the potential role of aspirin in cancer prevention and the recommendation for primary prevention in average-risk individuals (Figure 1).

If only CV benefits and harm (such as aspirin-induced bleeding) are considered, the reduction in CV events would be at least partially offset by the increase in major bleeds. However, if low-dose aspirin treatment also causes a hypothetical 10% reduction in overall cancer incidence, then the net effect of therapy becomes clearly favorable for a much larger population [1]. Therefore, future practice guidelines for aspirin prophylaxis may also consider prevention of cancer and not only the benefits of aspirin for the prevention of vascular disease.

Before recommending the use of low-dose of aspirin in the prevention of cancer it is necessary to shed light in the mechanism of action of aspirin in cancer prevention. The finding that low-doses of aspirin are efficacious both in the prevention of vascular events and cancer suggests that the two pathological conditions may have a common mechanism of action. The most plausible hypothesis is that an abnormal repair process mediated by platelet activation at distinct sites of tissue damage plays a key mechanism involved in atherothrombosis and cancer. Proof-of-concept studies will be carried out and translation research strategies will be used to validate this hypothesis. If it will be confirmed, it will open the way to the rational use of traditional antiplatelet drugs, such as aspirin and clopidogrel, and possibly novel antiplatelet agents for cancer chemoprevention. Moreover, the results of these studies will be useful for the development of personalized treatments to cancer chemoprevention.

## 3. Mechanism of Action of Aspirin

Aspirin shares with the other NSAIDs a common mechanism of action, *i.e.* the capacity to reduce prostanoid generation by inhibiting the activity of COX-isozymes. Prostanoids are biologically active derivatives of arachidonic acid (AA) released from membrane phospholipids through the activity of phospholipases [44,45]. 

Two isoforms of COX exist, named COX-1 and COX-2 [46]. Both COX-isozymes are differently regulated catalytically, transcriptionally and post-transcriptionally, but they share the same catalytic activities: i) cyclooxygenase activity which oxidizes AA to prostaglandin (PG)G_2_ and ii) peroxidase activity which reduces PGG_2_ to the unstable endoperoxide PGH_2_. Importantly, the two isozymes are homodimers and each monomer is able to bind the substrate, AA. However, one monomer acts as an allosteric subunit (regulatory), by its capacity to transform the partner monomer into the catalytic one transforming AA to PGG_2_; then PGG_2_ is transformed to PGH_2_ by the peroxidase activity of COX-1 and COX-2 [47,48,49]. PGH_2_ is subsequently metabolized by terminal synthases into the biologically active prostanoids, *i.e.* prostaglandin (PG)E_2_, PGF_2__α_, PGD_2_, prostacyclin(PGI_2_) and thromboxane (TX)A_2_.

COX-1 gene is considered a “housekeeping gene” and the protein is highly expressed in platelets where it is responsible for the generation of TXA_2_, which promotes platelet activation and aggregation, vasocostriction and proliferation of vascular smooth muscle cells [45,50]. In addition, COX-1 is highly expressed in gastric epithelial cells, where it plays an important role in cytoprotection through the generation of prostanoids, such as PGE_2 _[45,50]. In contrast, COX-2 gene, a primary response one with many regulatory sites [51], is constitutively expressed in some tissues in physiologic conditions, such as endothelium, kidney and brain, and in pathological conditions, such as in cancer [52]. In cancer cells, the major prostanoid produced through COX-2 is PGE_2_, which plays important roles in modulating motility, proliferation and resistance to apoptosis [53,54].

Aspirin, but not other NSAIDs, is able to cause an irreversible inactivation of COX isozymes through the acetylation of a specific serine moiety (Ser529 of COX-1 and Ser516 of COX-2) [55]. In detail, aspirin binds to one monomer of COX-1 and COX-2 by the interaction with Arg120 residue and modifies covalently COX isozymes acetylating Ser529 (in COX-1) and Ser516 (in COX-2); the acetylated monomer becomes the allosteric subunit, and the partner monomer becomes the catalytic monomer [56,57]. Acetylation of the allosteric subunit of COX-1 by aspirin causes an irreversible inhibition of the COX activity and in turn of the generation of PGG_2_ from AA. The acetylated COX-2 is not able to form PGG_2_, but it generates 15R-hydroxyeicosapentaenoic acid (15R-HETE) from AA [58]. *in vitro* studies have shown that 15R-HETE represents a substrate of 5-lipoxygenase (5-LOX), which metabolizes it into *epi*-lipoxins (LXs), characterized by anti-proliferative and anti-inflammatory actions [59,60,61]. However, convincing evidence that these lipid mediators triggered by aspirin are generated *in vivo* in humans are lacking. It is noteworthy to consider that systemic concentrations of aspirin, reached after dosing with low-doses, are inadequate to significantly acetylate COX-2 [5,62] (Table 2). It can be hypothesized that local aspirin concentrations obtained in the digestive system, including colon and esophagus, after dosing with the drug, are sufficiently high to acetylate epithelial COX-isozymes. However, it is necessary to perform further studies, using appropriate biomarkers, in order to get the last word on this issue.

**Table 2:** Pharmacodynamic and pharmacokinetics characteristics of aspirin.

**Chemical structure**	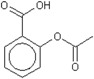 acetylsalicylic acid
**IC_50_ (human whole blood assays *in vitro* ); **mean(95% confidence interval, CI)	COX-1:7.9 (4.4–14)μM [65] COX-2: >5000μM [65]
**Oral bioavailability (%)**	49.2 (40 mg single dose) [68] 48.2 (325 mg single dose) [68] 50.7 (325 mg for 7 days) [68] Approx 30% (enteric coated aspirin) [72,73]
**Time to maximal plasma concentration**	30–40 minutes [74] 3–4 h (enteric coated aspirin)[72]
**Maximal plasma concentration ** **(** **μ** **M; mean±SD)**	acetylsalicylic acid: **antiplatelet dose(75**–**100mg/day):** 0.52±0.12 μM (controlled release) [5] 7.3±2μM (solution) [5] **analgesic dose (325, 600 mg):**25±12(68),80±20μM [75] **antiinflammatory dose(1.2g): **144±22 μM [75] **salicylic acid:** **antiplatelet dose(75**–**100mg/day):** 4.9±2.5 μM (controlled release) [5] 14.4±5 μM (solution) [5] **analgesic dose**(600mg/day)**: **297±96 μM [75] **anti-inflammatory dose:** (1.2 g/day), 585±164 μM [75] (5.85–6.2 g/day),1800–2300μM [76,77]
**Half-life (minutes)**	15–20 [74]
**Volume of distribution (l)**	21.2 (40 mg single dose) [68] 18.4 (325 mg single dose) [68] 19.2 (325 mg daily for 7 days) [68]
**Bound in plasma (%)**	80–90%[74,78]

### 3.1. Pharmacology of Aspirin

Pharmacokinetics and pharmacodynamics features of aspirin are reported in Table 2. Aspirin has a short half-life when administered *in vivo* and it is rapidly inactivated by plasma and tissue esterases to salicylic acid which is a weak inhibitor of COXs (in the millimolar range) [62,63,64]. The inhibitory effects of aspirin towards platelet COX-1 and monocyte COX-2 have been assessed in human whole blood *in vitro* and it has been found that aspirin is >100-fold more potent in inhibiting platelet COX-1 than monocyte COX-2 [62,63,64,65] (Table 2).

When administered at low-doses (75–100 mg daily), aspirin is able to cause an almost complete inhibition of the capacity of platelet COX-1 to generate TXA_2_ [55,66]. Thus, the maximal inhibition of TXA_2_-dependent platelet function occurs at low-doses of aspirin. Due to irreversible inhibition of COX-1 and to the fact that platelets have a limited capacity of de novo protein synthesis [67], the profound inhibitory effect of platelet function by aspirin persists throughout dose interval (i.e. 24 h).

The major part of the inhibitory effect of platelet COX-1 by the oral administration of low-dose aspirin occurs in the presystemic circulation where the drug reaches higher concentrations [55,68]. The drug undergoes the first-pass effect and its systemic concentration is quite low (after dosing with 75 mg, the Cmax is approximately 7 μM) (Table 2) (5), and insufficient to completely suppress platelet COX-1 activity. When aspirin is administered as sustained-release, microencapsulated preparations, the systemic concentration of the drug is negligible, *i.e.* less than 1 μM, and unable to affect COX-1 and COX-2 [5]. 

The impact of low-dose aspirin, administered once daily, on COX-2 activity *in vivo* is quite marginal. In fact, the systemic concentrations of the drug are inadequate to significantly affect this COX-isoform (Table 2) and even if a marginal inhibition may occur it should be almost completely recovered within a few hours in a nucleated cell [62,69,70,71]. Altogether the pharmacokinetics and pharmacodynamics features of low-dose aspirin support the notion that the drug is acting mainly by affecting platelet function as a consequence of COX-1 inhibition. At higher doses, aspirin may affect COX-2 in a dose-dependent fashion.

### 3.2. Clinical consequences of COX inhibition by aspirin

Aspirin is the only NSAID used as antithrombotic agent due to its unique pharmacodynamic features [12,55,70]. In fact, the almost complete and persistent inhibition of platelet COX-1 activity is a central mechanism for the prevention of atherothrombosis. This can be obtained with aspirin at low-doses [66,71]. In contrast, the inhibition of platelet COX-1 by the administration of nonaspirin NSAIDs, which are reversible inhibitors of COXs, is short-lasting, because it decreases in parallel with the reduction of their systemic concentration. The only nonaspirin NSAID which may cause an aspirin-like effect in respect to platelets, at least in some individuals, is naproxen when administered at high-doses (500 mg BID) because of its long half-life (*i.e*. 17 hours) and potent inhibitory effect towards COX-1 [69,74]. Differently from low-dose aspirin, naproxen profoundly affects COX-2 and extraplatelet sources of COX-1, such as the gastrointestinal tract. The inhibition of vascular COX-2-dependent prostacyclin by naproxen [69,79] may explain the fact that its administration is not associated with the prevention of vascular events [80]. In fact, prostacyclin is an important vasoprotective mediator [81]. The inhibition of COX-1-dependent PGE_2_ in the gastrointestinal tract by naproxen together with its antiplatelet effect seems to be the mechanisms involved in naproxen gastrointestinal toxicity [50]. 

Aspirin use is associated with reduction of non-fatal cardiovascular disease events (25 to 30%) and fatal events (10 to 15%) in RCTs of secondary prevention,without an apparent dose-dependent effect. In fact, the maximal benefit has been detected at low-doses (75–150 mg daily) [12]. At higher doses, a trend towards a reduced efficacy [12] was shown, possibly due to the inhibition of COX-2-dependent prostacyclin [71,81].

At higher doses (>325 mg daily), aspirin is used as an efficacious analgesic and antiinflammatory agent [74] and it acts by affecting COX-2-dependent prostanoids in inflammatory cells and spinal cord [82,83]. At antiinflammatory doses of aspirin (> 1,000 mg/day, Table 2) circulating levels of salicylic acid might contribute to inhibit COX-2 activity [62,63,64,75,76,77].

Long-term therapy with low-medium doses of aspirin (75–325 mg daily) is associated with an almost 2-fold increase in the risk of upper gastrointestinal bleeding compared with non-use, as demonstrated by observational studies [84,85] and by a meta-analysis of RCTs in high-risk patients [12]. Similar to nonaspirin NSAIDs, the risk of upper gastrointestinal bleeding increases (by>4-fold) at higher doses of aspirin, compared to aspirin non-use [84].The most plausible interpretation of these findings is that upper gastrointestinal bleeding associated with the use of low-dose aspirin is mainly due to its inhibitory effect on platelet function [50]; at higher doses, the additional inhibitory effect of the drug on the biosynthesis of cytoprotective prostanoids, in the GI tract, contributes to higher risk of upper gastrointestinal bleeding.

## 4. Evidences for COX-Dependent Mechanisms of the Antitumoral Effects of Aspirin

As we explained above, a large body of evidence indicates that aspirin can protect towards different types of cancer, in particular colorectal cancer [1], however the inhibitory mechanisms underlying this effect remain controversial. A role of the antiplatelet effect of aspirin in its anti-cancer effect is supported by some indirect evidences: i) similarly to that found for cardiovascular prevention by aspirin, an apparent saturability of the antitumor effects occurs at low-doses given once daily, in long-term analyses of cardiovascular randomized RCTs [3,4] and RCTs of adenoma recurrence [7,8,9,10], as well as in the vast majority of observational studies performed in different settings and with different methodology [11]; ii) one of the cardiovascular RCTs in which the chemopreventive effect of aspirin was detected on long-term follow-up (*i.e*. Thrombosis Prevention Trial, TPT) [2] involved the administration of a controlled-release formulation of aspirin (75 mg) (with negligible systemic bioavailability) [5].

Based on the knowledge of the pharmacology of aspirin, it has been proposed that low-dose aspirin and high-dose aspirin may affect different steps of colon tumorigenesis. Low-dose aspirin may inhibit relatively early events in the progression from normal mucosa to adenoma by blocking selectively platelet COX-1 activity and in turn platelet-dependent induction of cell transformation (possibly mediated by upregulation of COX-2 in stromal cells) [50]. In other words, low-dose aspirin might counteract the upregulation of COX-2 expression possibly induced by platelets in stromal cells and intestinal epithelial cells. Higher doses of aspirin might affect also directly COX-2 activity once it is expressed in intestinal adenomas and this may translate into reduction of advanced lesion progression and metastasis. Low-dose aspirin may also affect metastasis [39] by inhibiting platelet activation which is considered an important phenomenon in tumor spreading [86].

Recently, Ulrych and collaborators have shown that aspirin, both *in vitro* (at micromolar concentrations) and *ex vivo* [after dosing with a single analgesic dose (500 mg) or after the administration of an anti-platelet dose of 100 mg day, for 3 days], inhibits the release of sphingosine-1-phosphate (S1P) from human platelets even after stimulation with the potent peptide agonist of the thrombin receptor PAR-1(protease-activated receptor-1) [87]. The effect of aspirin was mediated by the inhibition of platelet COX-1-dependent TXA_2 _generation. In fact, formation and release of S1P from platelets is dependent on the activation of the TXA_2_ receptor (TP). S1P plays key roles as regulatory molecule in cancer development [88,89], by the promotion of cell proliferation, survival and regulation of angiogenesis, thus suggesting its implication in tumorigenesis. In humans, S1P is a natural constituent of plasma and is generated from sphingosine (Sph) via sphingosine kinase (SPHK), of which two isoforms (SPHK1 and -2) are known [90]. Platelets generate and store high amounts of S1P which is released upon stimulation with activators of protein kinase C (PKC) such as thrombin and TXA_2_ [90]. SPHK is highly active in platelets, which, however, lack the ability to synthesize the substrate Sph [90]. Thus, uptake of extracellular Sph and subsequent phosphorylation to S1P has been proposed as the primary mechanism of S1P formation in platelets [91+. As platelets lack the S1P-degrading enzyme S1P lyase, S1P accumulates intracellularly and large amounts are released upon platelet activation [91]. 

In one study, it has been detected the inhibition of PGE_2_ in rectal biopsies performed after 1 month of treatment with three aspirin doses (81, 325, and 650 mg) or placebo [92]. Unexpectedly, also the 81-mg daily aspirin dose suppressed PGE_2 _levels to an equivalent extent as did the 650-mg dose. In another study it has been shown that the treatment with 81 mg of aspirin per day for three months reduced mucosal PGE_2_ formation and transforming growth factor α (TGF-α) expression in normal appearing rectal mucosa from individuals with a history of adenomatous polyps [93]. These findings may indicate that low-dose aspirin is sufficient at attenuating PGE_2_ levels in the GI microenvironment. However, it has been claimed that contaminating platelets in rectal biopsies might have contributed significantly to the generation of prostanoids by rectal mucosa and thus explaining the efficacy of low-dose aspirin to affect prostanoid generation in this setting. Therefore, further studies using more appropriate methodologies should be performed to definitively clarify whether low-dose aspirin affects COX-1 activity in the gastrointestinal tract. Moreover, these studies should clarify the possible contribution of aspirin's effect on platelet COX-1 activity in the apparent reduction of prostanoid generation in rectal mucosa. These studies are urgently required to address the current uncertainty concerning the optimal dose of aspirin and dosing regimen for cancer prevention.

## 5. Evidences for COX-Independent Mechanisms of the Antitumoral Effects of Aspirin

**Figure 2:**
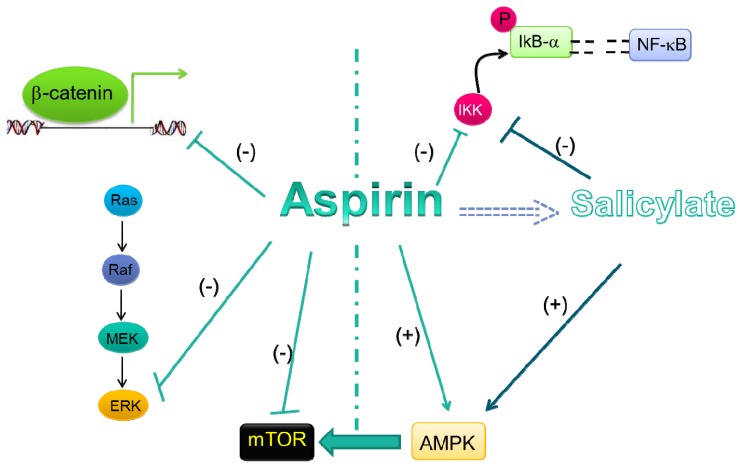
COX-independent mechanisms of the antitumoral effects of high-doses of aspirin and salicylate.

Several COX-independent mechanisms of aspirin have been reported that might contribute to its chemopreventive effects in tumorigenesis [94]. Most of these effects have been found * in vitro* using elevated concentrations of aspirin (often in the millimolar range) which cannot be obtained in the systemic circulation after dosing with low-doses of the drug, but might require the use of very high anti-inflammatory doses of aspirin (Table 2). Under these experimental conditions, different COX-independent antitumor effects have been reported (summarized in Figure 2). However, it is important to underline that no convincing piece of evidence has been obtained to demonstrate that these mechanisms are operative *in vivo* in particular after dosing with low-doses of aspirin which have been associated with the chemopreventive benefit of aspirin in RCTs.

The COX-independent pathways which have been shown to be altered by high concentrations of aspirin involve: i) the inhibition of pro-tumoral signalings (such as Wnt/β-catenin, ERK, NF-kb and mTOR); and ii) the activation of anti-tumor signaling (such as AMPK pathway). At high concentrations of aspirin, it is possible that the formation of salicylate from aspirin (Table 2) could contribute to anti-tumor effects.

### 5.1. Wnt/β-Catenin Pathway

The activation of Wnt/β-catenin pathway is the first step in almost all colorectal cancers (CRC) and occurs through the cytoplasmic accumulation of free β-catenin [95]. In most cases, Wnt/β-catenin pathway activation is a consequence of a mutation in the adenomatous polyposis coli (APC) gene [96]. This leads to a truncated form of APC protein which has lost its function to be a part of the β-catenin destruction complex. This translates into reduced β-catenin degradation associated with abnormally high level of cytoplasmic β-catenin. The stabilized β-catenin translocates to the nucleus where it binds members of the T-cell factor (Tcf)/lymphoid enhancer factor (Lef) family of transcription factors, and activates the transcription of Wnt target genes such as cyclin D and Myc [97]. Bos *et al.* [98] investigated the effects of aspirin on the oncogenic Wnt/β-catenin pathway activity in CRC cell lines and observed that aspirin inhibits the Wnt/β-catenin pathway by stimulating the phosphorylation with the consequent degradation of β-catenin. In fact, in a range of millimolar concentrations (which cannot be reached after dosing with aspirin, even at high antiiflammatory doses, Table 2), aspirin inhibited protein phosphatase 2A (PP2A) enzymatic activity, a protein which influences the phosphorylation status of β-catenin. This effect of aspirin acting through PP2A inhibition causes an increase of the levels of phosphorylated β-catenin and its degradation which translates into a reduction of Wnt/βcatenin pathway activity.

### 5.2. ERK Signaling

It has been shown that supratherapeutic concentrations ofNSAIDs, including aspirin, may modulate COX-independent biological responses *in vitro* through alterations of the activity of kinases involved in different signaling pathways, such as extracellular signal-regulated kinases (i.e. ERK1/2) [99] (Figure 2). The ERK pathway is activated in a variety of cell types by diverse extracellular stimuli and its activation leads to the phosphorylation of various downstream substrates involved in a multitude of cellular responses such as cell proliferation, cell differentiation, cell survival, and cell motility [99]. Aberrant activation of the ERK pathway has been shown to be an essential feature common to many types of human tumors. Interestingly, Pan *et al.* [100] have recently shown that several NSAIDs, including aspirin, suppress the ERK-mediated signaling by inhibiting the binding of Ras oncogene and c-Raf kinase that leads to constitutive activation of ERK, a distinct event of tumor progression.

### 5.3. NF-κB Signal Transduction Pathway

Several studies have shown that modulation of the NF-κB signal transduction pathway is a key mechanism for the pro-apoptotic activity of aspirin and other NSAIDs [101,102] (Figure 2). The NF-kB transcription factor generally exists as a heterodimer bound in the cytoplasm by the inhibitor protein IκB. Following cellular stimulation by specific inducers, IκB is phosphorylated by the I-κB kinase (IKK) complex and then degraded by the ubiquitin-proteasome machinery [103]. Subsequently, NF-kB translocates to the nucleus where it regulates transcription of its target genes, which include many that control cell growth. It has been shown that aspirin, as well as sodium salicylate, inhibits IKK-β activity *in vitro* at millimolar concentration [104] by binding to IKK-β, thus, competing with ATP for the binding to the kinase, an event necessary to phosphorylate IkB [105,106]. These concentrations of aspirin cannot be reached even after dosing with anti-inflammatory doses of the drug (Table 2). In contrast, these levels of salicylate are detected in the systemic circulation when aspirin is given at high doses (Table 2) and they could affect this COX-independent pathway *in vivo* .

### 5.4. AMPK/mTORsignaling

AMP-activated protein kinase (AMPK) is a critical cellular energy sensor, which is activated under stresses such as hypoxia, ischemia, glucose deprivation and exercise [107]. Activation of AMPK stimulates fatty acid oxidation to generate more ATP to cope with acute energy demand and inhibits anabolic processes that consume ATP. Recently great attention has been drawn to link AMPK and cancer. In fact, AMPK plays an important role in the regulation of a variety of pathways implicated in cell tumor progression [108,109], such as p53, fatty acid synthase (FASN) and the mechanistic target of rapamycin (mTOR), which controls cell survival and regulation of metabolism [110,111] and it is an important signaling in cancer development, including CRC. Din *et al.* [111] investigated the effects of aspirin on AMPK/mTOR signaling. In CRC cells, millimolar concentration of aspirin reduced mTOR signaling by inhibiting the mTOR effectors S6K1 and 4E-BP1. Furthermore, aspirin changed nucleotide ratios and activated AMPK in CRC cells. The authors gave a demonstration of the mTOR inhibition by aspirin also *in vivo* in CRC patients treated with an analgesic dose of 600 mg of aspirin, once daily for one week. In the rectal mucosa of these patients they found a reduced phosphorylation of S6K1 and S6, the key effectors of mTOR signaling. 

Together with this observation of aspirin effects on mTOR/AMPK signaling, Hawley and collaborators [112] described, for the first time, that salicylate [at concentration of 1-3 mM which can be reached after dosing with anti-inflammatory doses of aspirin, Table 2] can directly activate AMPK, primarily by inhibiting dephosphorylation at residue threonine (Thr)-172. Going deeper in the mechanism of action of salicylate in AMPK activation, the authors suggest that salicylate protects AMPK against Thr172 dephosphorylation and inactivation by the inhibition of protein phosphatase-2Ca activity.

### 5.5. Acetylation of Other Proteins by Aspirin

Aspirin has been shown to acetylate several proteins and biomolecules such as hemoglobin, DNA, RNA and histones, as well as several plasma constituents, including hormones and enzymes [113,114]. Recently it has been shown that in the human breast cancer cell line MDA-MB-231 aspirin at 100 μM acetylates the tumor suppressor protein p53 [115]. p53 is a key regulator of apoptosis, and is acetylated at several defined sites by cellular acetyltransferases in response to various stresses, including DNA damage [116]. Increased acetylation of p53 by aspirin correlated with increased p53 DNA binding activity and the expression of two of its target genes, p21^CIP1^, a protein involved in cell cycle arrest, and Bax, a mitochondrial pro-apoptotic protein [115]. Moreover, aspirin at 100-300 μM acetylates multiple proteins at lysine residues in both HCT-116 and HT-29 colon cancer cells. Among the proteins acetylated by aspirin, it is worth mention in glucose 6 phosphate dehydrogenase (G6PD), which regulates ribonucletide biosynthesis, because its acetylated form was associated with the inhibition of the activity [117]. 

These results are interesting but it seems unlikely that they play a role in the antitumorigenic effects of low-dose aspirin due to the low-concentrations of the drug reached in the systemic circulation (Table 2) and to the once-a-day administration which should lead to a short-lasting inhibitory effect in a nucleated cell. Differently, the possible occurrence of the acetylation of COX-independent targets after dosing with repeated daily, high anti-inflammatory doses of aspirin cannot be excluded but it remains to be verified. However, the finding that the apparent maximal benefit of aspirin as chemopreventive agent was detected after dosing with low-doses, once-a-day seems to eclipse the importance of this extra-COX-1 acetylation activity of aspirin *in vivo* .

## 6. Conclusions

A large body of clinical evidence supports the protective action of aspirin as chemopreventive agent for different types of cancer, in particular colorectal cancer [1]. The accumulation of different indirect evidences leads to hypothesize that the antiplatelet effect of aspirin is a central mechanism for its antitumor effect [1,50,62]. It is quite persuasive the finding of an apparent maximal chemopreventive efficacy against cancer and atherothrombosis by low-dose aspirin [1]. At low-doses given every 24 h, aspirin is acting by a complete and persistent inhibition of COX-1 in platelets(in the pre-systemic circulation) [68], while causing a limited and rapidly reversible inhibitory effect on COX-2 and/or COX-1 expressed in nucleated cells [55]. 

The role of platelets has been recognized from a long time in the process of spreading of neoplastic cells to other organs or to lymph nodes far from the primary tumor(called metastatic disease) [86]. However, the recent clinical findings with aspirin suggest that platelets play a role in early phases of tumorigenesis [38,50,62]. Platelets are activated in intestinal tumorigenesis in humans [13,14], and they may contribute to tumor development through the release of different constituents, such as growth and angiogenic factors and lipid mediators [86]. These platelet products may trigger the release of high levels of growth factors from stromal cells, possibly as a consequence of COX-2 overexpression. Stromal activation, then, may lead to epithelial cell transformation, at least in part by inducing an aberrant COX-2 expression in epithelial cells [50,54]. Here COX-2-derived prostanoids cause cell proliferation and the accumulation of mutations, as a consequence of apoptosis inhibition [53,54,118,119]. The proof of concept of this hypothesis is coming from the data obtained in animal model of intestinal tumorigenesis showing that loss of either COX-1 or COX-2 genes blocks profoundly and similarly intestinal polyposis [120,121], thus suggesting that the two COX-isozymes operate sequentially and it is plausible that COX-1 acts upstream of COX-2.

Extensive translational medicine research will be performed in the next five years to confirm the platelet-mediated hypothesis of colon tumorigenesis. Importantly, these studies will address the current uncertainty concerning the optimal aspirin dose and dosing regimen for cancer prevention and the possible contribution of individual genetic cancer susceptibility to aspirin response [122]. This will lead to identify novel mechanisms of disease, novel therapeutic approaches in chemoprevention and the development of biomarkers for early diagnosis and individualized prevention.
